# Supplementary material for: Regional practice variation in induction of labor in the Netherlands: Does it matter? A multilevel analysis of the association between induction rates and perinatal and maternal outcomes
Source: PLoS One. 2023 Jun 8;18(6):e0286863. doi: 10.1371/journal.pone.0286863 (PMC10249899; doi:10.1371/journal.pone.0286863)
Supplement: S1 Table — (DOCX) [file pone.0286863.s001.docx]

| **S1 Overview of nulliparous term singleton vertex (NTSV) population in MCNs 2016-2018** | | | | | | |  | |  | |  | |
| --- | --- | --- | --- | --- | --- | --- | --- | --- | --- | --- | --- | --- |
|  | **2016** |  | **2017** |  | **2018** |  | | **Total** | |  | |  |
| **Number NTSV per gestational age** |  |  |  |  |  |  | |  | |  | |  |
| 37 wk | 4691 | 7.4% | 4527 | 7.3% | 4199 | 7.1% | | 13417 | | 7.3% | |  |
| 38 wk | 9391 | 14.8% | 9334 | 15.1% | 8746 | 14.7% | | 27471 | | 14.9% | |  |
| 39 wk | 15236 | 24.0% | 15107 | 24.5% | 14587 | 24.6% | | 44930 | | 24.4% | |  |
| 40 wk | 19417 | 30.6% | 18535 | 30.1% | 17964 | 30.3% | | 55916 | | 30.3% | |  |
| 41 wk | 13257 | 20.9% | 12718 | 20.6% | 12505 | 21.1% | | 38480 | | 20.9% | |  |
| 42 wk | 1466 | 2.3% | 1432 | 2.3% | 1310 | 2.2% | | 4149 | | 2.2% | |  |
| Total | 63458 |  | 61653 |  | 59311 |  | | 184422 | |  | |  |
| **NTSV in MCNs** |  |  |  |  |  |  | |  | |  | |  |
| number MCNs | 76 |  | 76 |  | 77 |  | | 77 | |  | |  |
| Mean (SD) number NTSV per MCN | 835.0 | (471.8) | 811.2 | (449.1) | 770.3 | (445.8) | | 2416.4 | | (1334.4) | |  |
