# Supplementary material for: Regional practice variation in induction of labor in the Netherlands: Does it matter? A multilevel analysis of the association between induction rates and perinatal and maternal outcomes
Source: PLoS One. 2023 Jun 8;18(6):e0286863. doi: 10.1371/journal.pone.0286863 (PMC10249899; doi:10.1371/journal.pone.0286863)
Supplement: S2 Table — (DOCX) [file pone.0286863.s002.docx]

| **S2 Start of labor per year in NTSV population** | | | | | | | | |
| --- | --- | --- | --- | --- | --- | --- | --- | --- |
|  | **2016** |  | **2017** |  | **2018** |  | **Total** |  |
|  | n= 63458 |  | n=61653 |  | n=59311 |  | n=184422 |  |
| **Start of labor** |  |  |  |  |  |  |  |  |
| spontaneous | 46892 | 73.9% | 45272 | 73.4% | 43466 | 73.3% | 135630 | 73.5% |
| Induction of labor (IOL) | 14755 | 23.3% | 14687 | 23.8% | 14143 | 23.8% | 43585 | 23.6% |
| Caesarean Section | 849 | 1.3% | 716 | 1.2% | 666 | 1.1% | 2231 | 1.2% |
| unknown | 962 | 1.5% | 978 | 1.6% | 1036 | 1.7% | 2976 | 1.6% |
| **Method IOL** |  |  |  |  |  |  |  |  |
| Amniotomy only | 2366 | 3.7% | 1947 | 3.2% | 1382 | 2.3% | 5695 | 3.1% |
| Prostaglandins | 2515 | 4.0% | 1872 | 3.0% | 1567 | 2.6% | 5954 | 3.2% |
| Oxytocin | 3558 | 5.6% | 3712 | 6.0% | 4011 | 6.8% | 11281 | 6.1% |
| Foley catheter | 6316 | 10.0% | 7156 | 11.6% | 7183 | 12.1% | 20655 | 11.2% |
| **IOL in week** |  |  |  |  |  |  |  |  |
| 37 | 1905 | 12.9% | 1816 | 12.4% | 1714 | 12.1% | 5435 | 12.5% |
| 38 | 3232 | 21.9% | 3482 | 23.7% | 3186 | 22.5% | 9900 | 22.7% |
| 39 | 2470 | 16.7% | 2561 | 17.4% | 2624 | 18.6% | 7655 | 17.6% |
| 40 | 2197 | 14.9% | 2162 | 14.7% | 2140 | 15.1% | 6499 | 14.9% |
| 41 | 3938 | 26.7% | 3667 | 25.0% | 3544 | 25.1% | 11149 | 25.6% |
| 42 | 1013 | 6.9% | 999 | 6.8% | 935 | 6.6% | 2947 | 6.8% |
| Total | 14755 |  | 14687 |  | 14143 |  | 43585 |  |
| **Mean (SD) gestational age** | 279.4 | (8.6) | 279.3 | (8.6) | 278.4 | (8.5) | 279.4 | (8.6) |
