# Supplementary material for: Regional practice variation in induction of labor in the Netherlands: Does it matter? A multilevel analysis of the association between induction rates and perinatal and maternal outcomes
Source: PLoS One. 2023 Jun 8;18(6):e0286863. doi: 10.1371/journal.pone.0286863 (PMC10249899; doi:10.1371/journal.pone.0286863)
Supplement: S3 Table — (DOCX) [file pone.0286863.s003.docx]

| **S3. NTSV population and pregnancy characteristics in MCNs with low, moderate and high IOL rate** | | | | | | |
| --- | --- | --- | --- | --- | --- | --- |
|  | **Q1 (low)**  **n= 52979** | | **Q2-3 (moderate)**  **n= 91638** | | **Q4 (high)**  **n= 39805** | |
| **Maternal sociodemographic factors** | | | | | | |
| **Maternal age** |  |  |  |  |  |  |
| ≤ 20 years | 1396 | 2.6% | 2601 | 2.8% | 1569 | 3.9% |
| 21-34 years | 44384 | 83.8% | 78090 | 85.2% | 33428 | 84.0% |
| ≥ 35 years | 7199 | 13.6% | 10947 | 11.9% | 4808 | 12.1% |
| Mean (SD) | 29.5 | SD 4.7 | 29.1 | SD 4.6 | 28.8 | SD 4.8 |
| **Socio-economic status** |  |  |  |  |  |  |
| Low (≤ p20) | 9204 | 17.5% | 16774 | 18.4% | 10400 | 26.3% |
| Intermediate (p21-p80) | 30439 | 57.7% | 57729 | 63.4% | 22474 | 56.8% |
| High (> p80) | 13075 | 24.8% | 16523 | 18.2% | 6677 | 16.9% |
| **Living in deprived area** | 9317 | 17.7% | 8963 | 9.8% | 6807 | 17.2% |
| **Non-Caucasian background** | 4788 | 9.1% | 7390 | 8.1% | 4401 | 11.2% |
| **Registered pregnancy complications** | | | | | | |
| Diabetes (DM or GDM) | 1723 | 3.3% | 3220 | 3.5% | 1903 | 4.8% |
| Suspected LGA | 832 | 1.6% | 1699 | 1.9% | 1176 | 3.0% |
| Suspected SGA | 1673 | 3.2% | 2824 | 3.1% | 1459 | 3.7% |
| Reduced fetal movements | 5140 | 9.7% | 8834 | 9.6% | 3928 | 9.9% |
| Hypertension/preeclampsia | 4011 | 7.6% | 7973 | 8.7% | 3170 | 8.0% |
| None of the above | 40759 | 76.9% | 69377 | 75.7% | 29266 | 73.5% |
| **Level of care and transfers during pregnancy** | | | | | | |
| Start in primary antenatal care | 49781 | 94.0% | 85518 | 93.3% | 36009 | 90.5% |
| Start in secondary antenatal care | 3147 | 5.9% | 6063 | 6.6% | 3727 | 9.4% |
| Transfer to secondary care during pregnancy (overall) | 14243 | 26.9% | 29696 | 32.4% | 14946 | 37.5% |
| ≤*36 wk* | *6565* | *12.4%* | *13937* | *15.2%* | *7139* | *17.9%* |
| *37-42 wk* | *6422* | *12.1%* | *12517* | *13.7%* | *5565* | *14.0%* |
| *timing unclear* | *1256* | *2.4%* | *3242* | *3.5%* | *2242* | *5.6%* |
| Start of labor in primary care | 35304 | 66.6% | 55441 | 60.5% | 20810 | 52.3% |
| *planned at home* | *6582* | *12.4%* | *11721* | *12.8%* | *2920* | *7.3%* |
| *planned in birth center* | *5329* | *10.1%* | *3653* | *4.0%* | *1749* | *4.4%* |
| *planned in hospital* | *16617* | *31.4%* | *31010* | *33.8%* | *11851* | *29.8%* |
| *planned place unknown* | *6776* | *12.8%* | *9057* | *9.9%* | *4290* | *10.8%* |
| Start of labor in secondary care | 17390 | 32.8% | 35759 | 39.0% | 18673 | 46.9% |
| **Neonatal characteristics** | | | | | | |
| Gestational age in days Mean (SD)* | 279.9 | SD 8.5 | 279.4 | SD 8.6 | 278.6 | SD 8.6 |
| Small for gestational age (< p3) | 1502 | 2.9% | 2645 | 2.9% | 1295 | 3.3% |
| Large for gestational age (> p97) | 882 | 1.7% | 1635 | 1.8% | 604 | 1.5% |
| Macrosomia (>4500 g)* | 687 | 1.3% | 1187 | 1.3% | 335 | 0.8% |
| Mean birthweight (SD)* | 3443 | SD 457 | 3434 | SD 460 | 3387 | SD 456 |
| Serious or lethal congenital malformation | 414 | 0.8% | 571 | 0.6% | 416 | 1.0% |
| * Differences in gestational age, birthweight and prevalence of macrosomia between IOL categories are statistically significant. Gestational age and birthweight are considered outcomes in case of IOL.  Missings in variables are not shown. Due to missings, numbers not always add up to the total number in Q1, Q2-3 and Q4. Percentages are calculated on valid numbers.  (G)DM: (Gestational) Diabetes Mellitus; LGA: Large for Gestational Age; SGA: Small for Gestational Age | | | | | | |
