# Supplementary material for: Regional practice variation in induction of labor in the Netherlands: Does it matter? A multilevel analysis of the association between induction rates and perinatal and maternal outcomes
Source: PLoS One. 2023 Jun 8;18(6):e0286863. doi: 10.1371/journal.pone.0286863 (PMC10249899; doi:10.1371/journal.pone.0286863)
Supplement: S1 Fig — (DOCX) [file pone.0286863.s004.docx]

**S1 Figure: MCN practice variation in IOL in nulliparous women with a term singleton pregnancy with a vertex presentation**

- Expected IOL is the number of IOL in a MCN after casemix correction for SES and ethnicity
- Deviation from expected is calculated as: (observed IOL – expected IOL)/(expected IOL)
